# Totally Extraperitoneal Repair of Grynfeltt’s Hernia: Easy Solution for a Rare Problem

**DOI:** 10.7759/cureus.74743

**Published:** 2024-11-29

**Authors:** MS Peethambaran, Rajkamal R Rajendran, Narayana G Murthy

**Affiliations:** 1 Gastrointestinal Surgery, Avitis Institute of Medical Sciences, Palakkad, IND; 2 Urology, Avitis Institute of Medical Sciences, Palakkad, IND

**Keywords:** grynfeltt hernia, grynfeltt-lesshaft hernia, laparoscopic hernia repair, laparoscopic repair, lumbar hernia repair, minimally access surgery, petit triangle, primary lumbar hernia, tep approach, totally extraperitoneal repair (tep)

## Abstract

Lumbar hernias are a rare form of abdomen wall hernias. As this is a rare disease, treatment options are not standardized. Most case reports, even recent ones, describe open techniques. A minimally invasive method, though feasible, is not done by most surgeons. Among the minimally invasive techniques, the transabdominal method is commonly performed. A totally extraperitoneal (TEP) approach is a good alternative, which can be done with minimal complications. Here, we describe a case report of a patient who had a superior lumbar hernia (Grynfeltt's) and inguinal hernia, who successfully underwent simultaneous TEP repair for the lumbar and inguinal hernia.

## Introduction

Lumbar hernias are very rare wall defects in the lumbar area accounting for less than 2% of abdominal hernias, as described by Ploneda-Valencia et al. [1]. In this kind of hernia, retroperitoneal (kidneys, urinary bladder, and ascending or descending colon) and intraperitoneal (small bowel, omentum, preperitoneal fat, stomach, spleen, etc.) elements bulge through a defect in the dorsal posterolateral abdominal wall. The region is divided into two spaces, the superior one known as the Grynfeltt-Lesshaft triangle, and the inferior space, also called Petit's triangle [2]. The Grynfeltt-Lesshaft triangle is an inverted triangle bounded superiorly by the 12th rib, medially by the erector spinae group of muscles, and laterally by the internal oblique muscle. Here, a case report of a 53-year-old male patient with a lumbar hernia treated laparoscopically is described.

## Case presentation

A 53-year-old male presented with swelling in the right inguinal and left loin region for the past three years. The swelling had an expansile impulse on coughing and straining. Past history revealed an open umbilical hernia mesh repair in 2010 and a left inguinal hernia Lichtenstein repair in 2016. Local examination revealed a left upper lumbar hernia. The patient also had a complete right inguinal hernia and a small left inguinal hernia.

**Figure 1:**
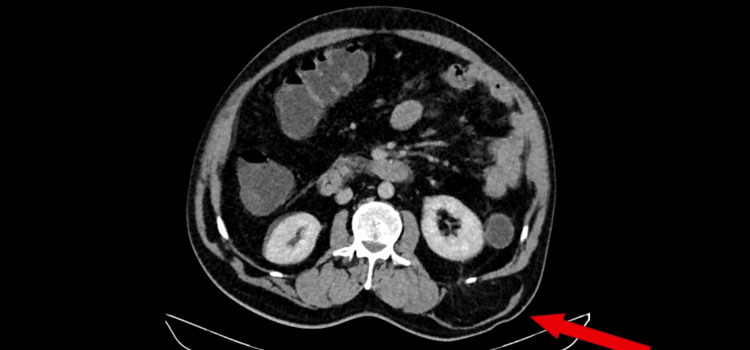
Left lumbar hernia defect seen in CT scan.

**Figure 2:**
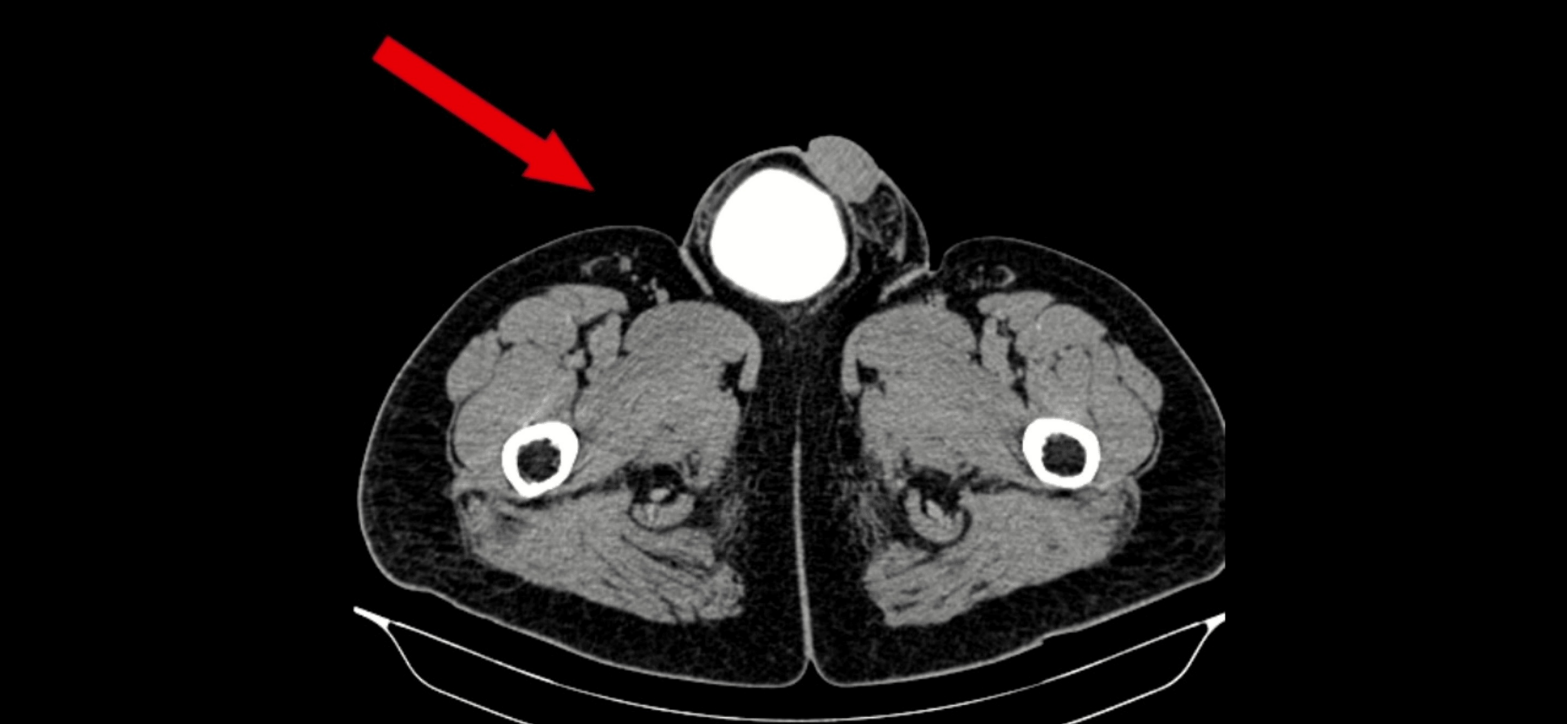
Right inguinoscrotal hernia with contrast seen in bladder within the scrotum.

Contrast-enhanced computed tomography (CECT) of the abdomen showed a right inguinal hernia with part of the urinary bladder reaching the base of the scrotum, a left inguinal hernia, and a left superior lumbar triangle defect with herniating adipose tissue. The CECT picture showed a lumbar defect with herniation of adipose tissue (Figure 1). The urogram phase of CECT showed a right inguinal hernia with contrast in the bladder (Figure 2).

Treatment options were discussed with the patient and the rarity of the disease was explained. The patient opted for simultaneous repair of all hernias if possible by minimal access technique. Different surgical options like open repair of lumbar hernia with bilateral totally extraperitoneal (TEP) inguinal repair, transabdominal preperitoneal (TAPP) repair for the lumbar hernia followed by TAPP/TEP repair for bilateral inguinal hernia, and TEP repair for both lumbar as well as bilateral inguinal hernias were considered. Finally, it was decided to proceed with both inguinal as well as lumbar hernia surgeries in a single setting using a minimally invasive approach.

Since the lumbar and inguinal hernia repair requires different patient positions, there is a chance for cross-contamination in the TAPP procedure. Hence it was decided to try a TEP approach for both the lumbar and groin hernias. Surgery was done under general anesthesia. Initially, the patient was placed in the right lateral kidney position with a table break to open up the retroperitoneal space between the 12th rib and the iliac crest.

**Figure 3:**
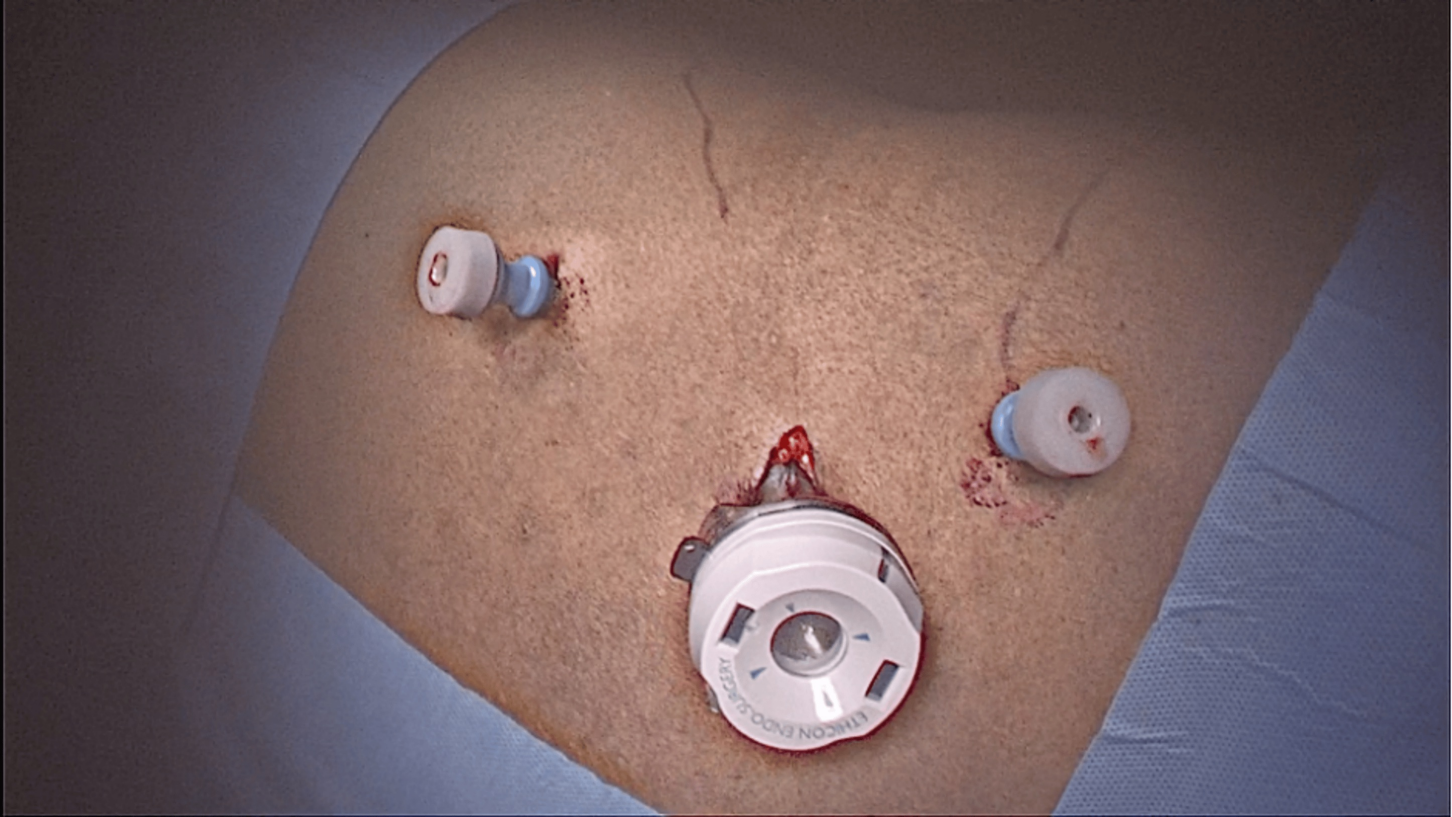
Port position.

An incision was made midway between the coastal margin and the iliac crest 1 cm lateral to the anterior axillary line for the first port. The abdomen wall muscles were separated under vision till the transversalis fascia. Trocar was carefully inserted and further dissection was done to create the space with a zero-degree telescope. Two 5 mm working ports, one below the costal margin and one above the iliac crest, were inserted under vision (Figure 3).

**Figure 4:**
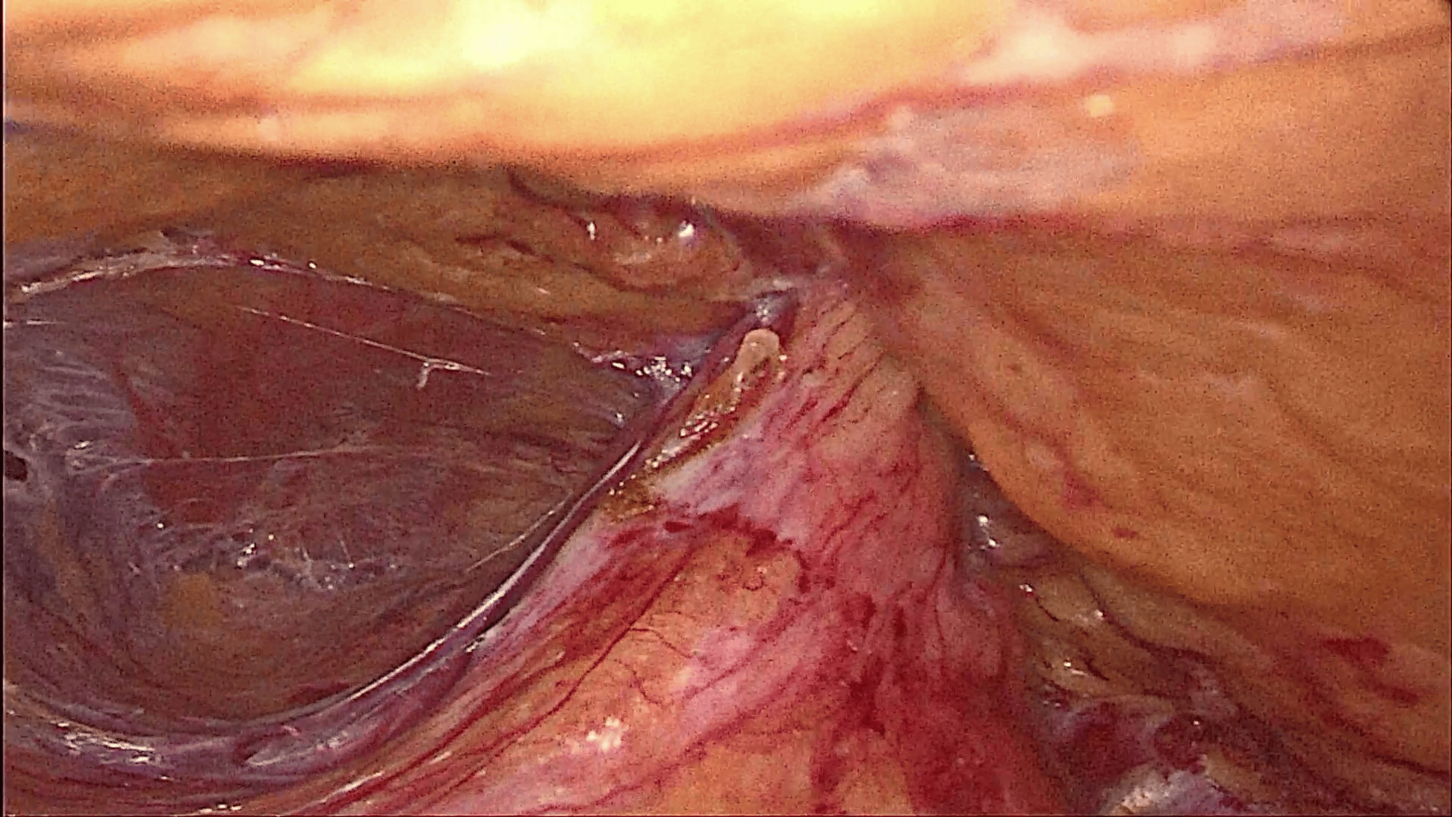
Hernial sac identified.

**Figure 5:**
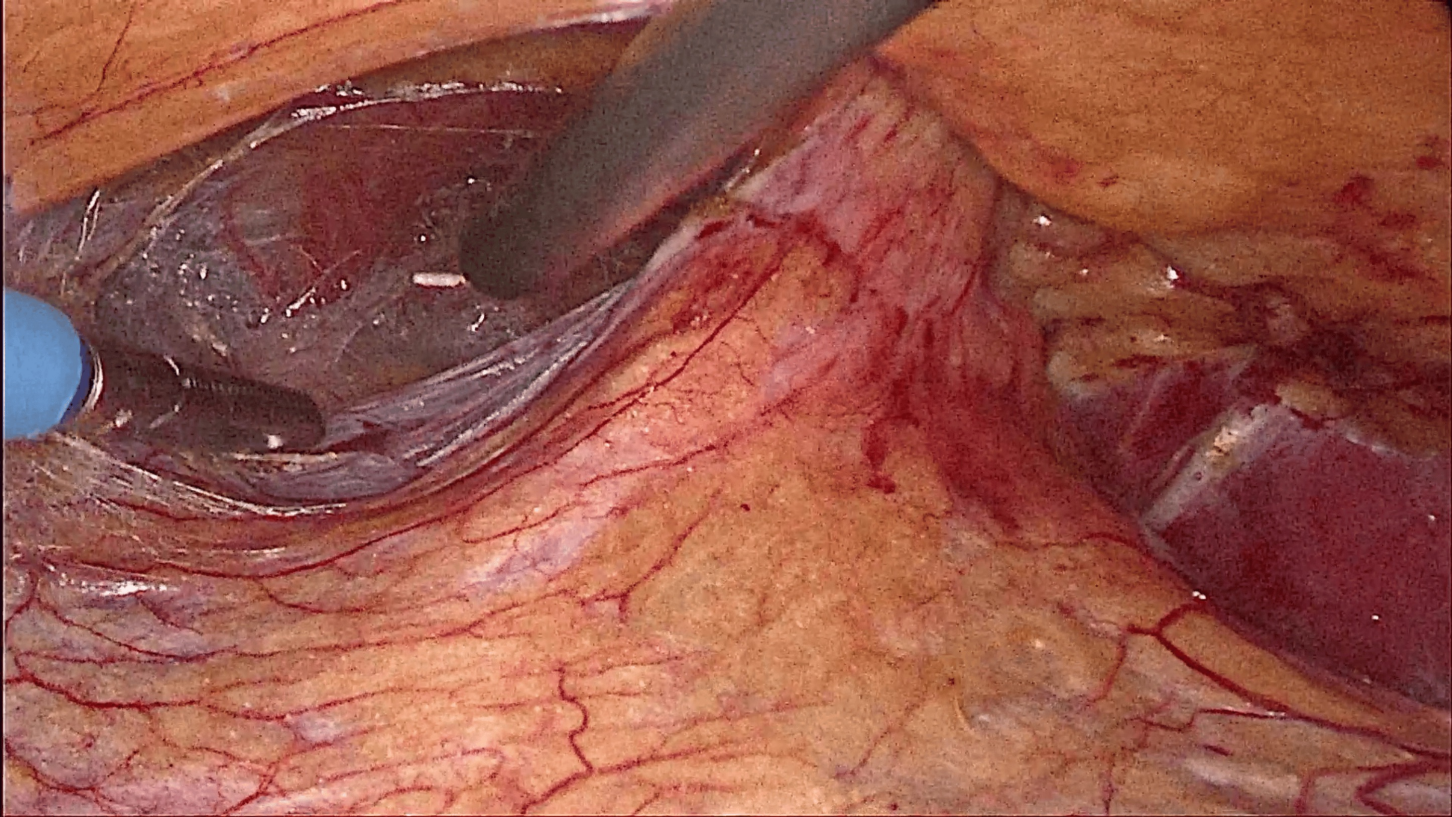
Circumferential dissection done around the sac.

**Figure 6:**
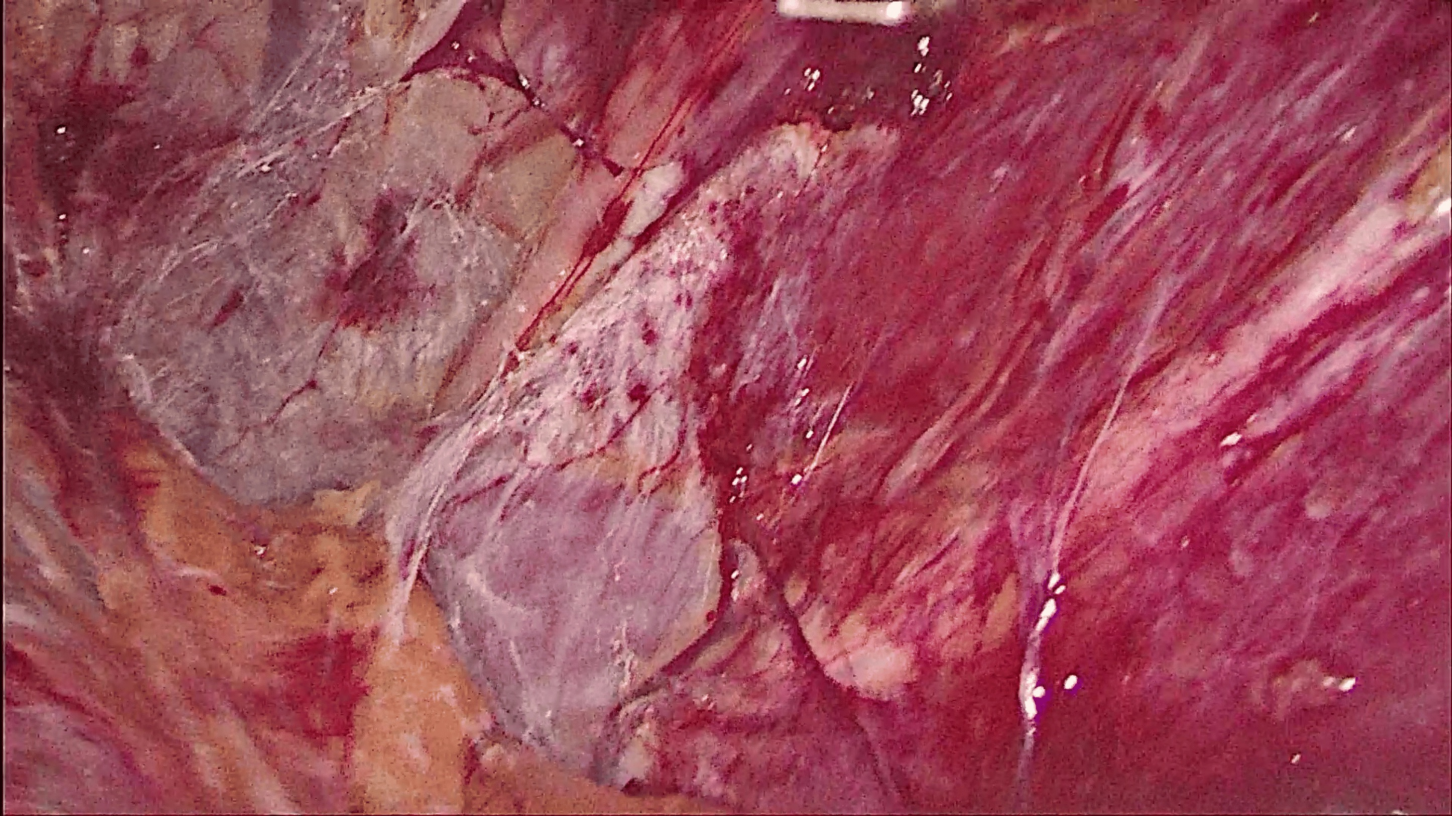
Lumbar nerves exposed.

**Figure 7:**
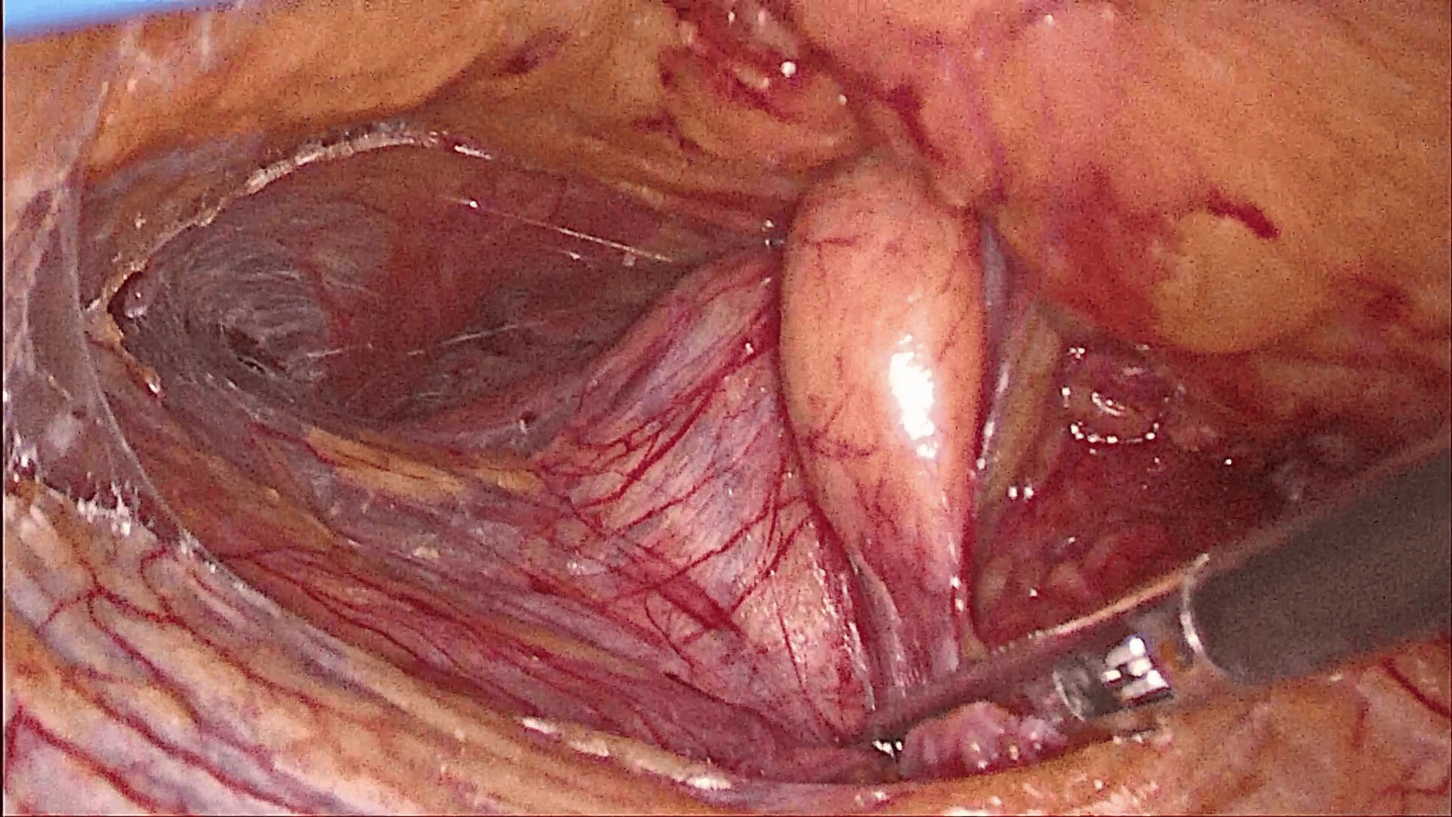
Reducing the contents.

**Figure 8:**
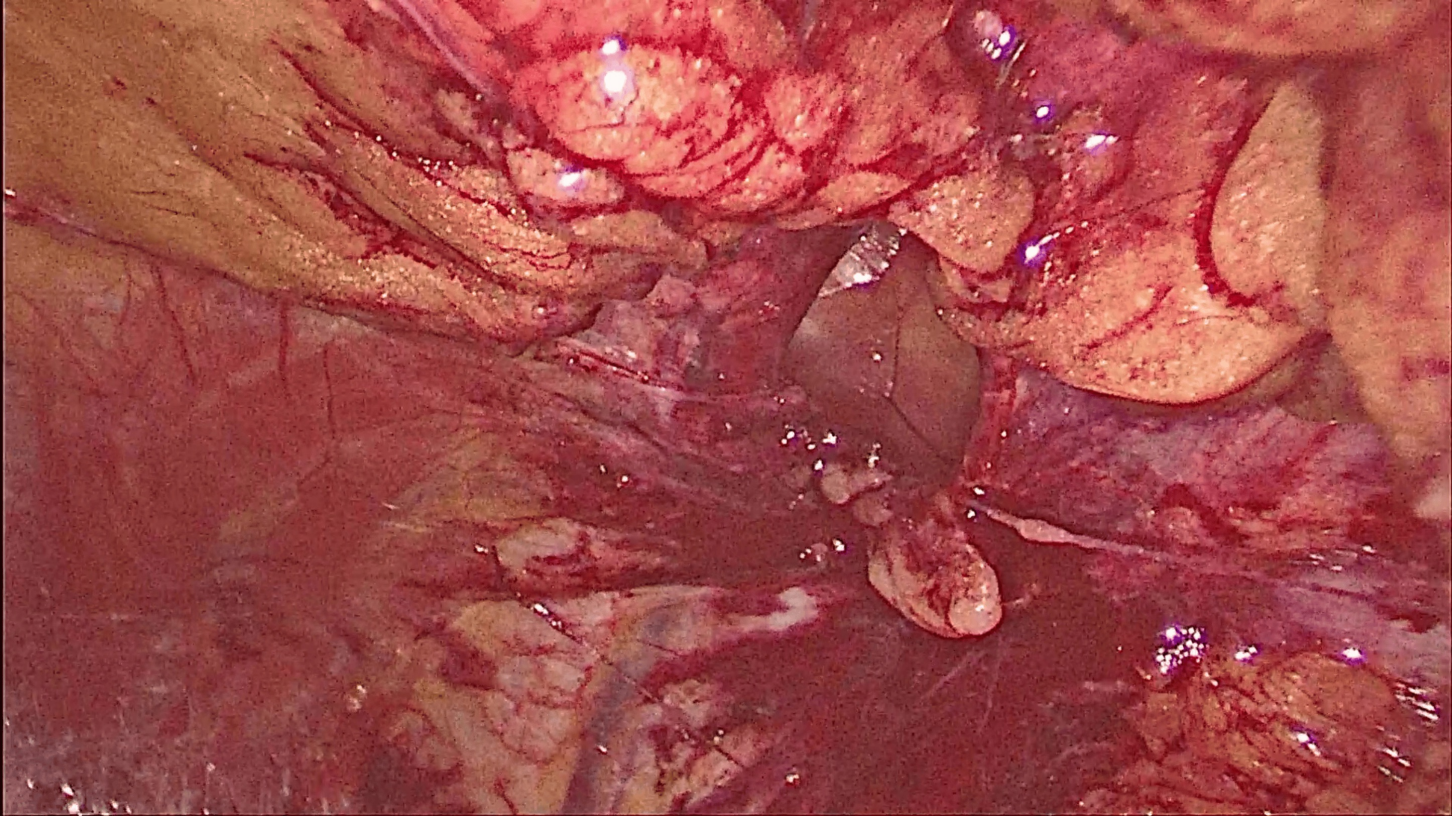
Defect after reduction of contents.

Further dissection was done till adequate space was created. Superiorly, the dissection extended into the rib cage, inferiorly to the pelvis, anteriorly to the midaxillary line, and posteriorly exposing the psoas muscles. The hernial sac was identified and the contents were reduced (Figures 4-8).

**Figure 9:**
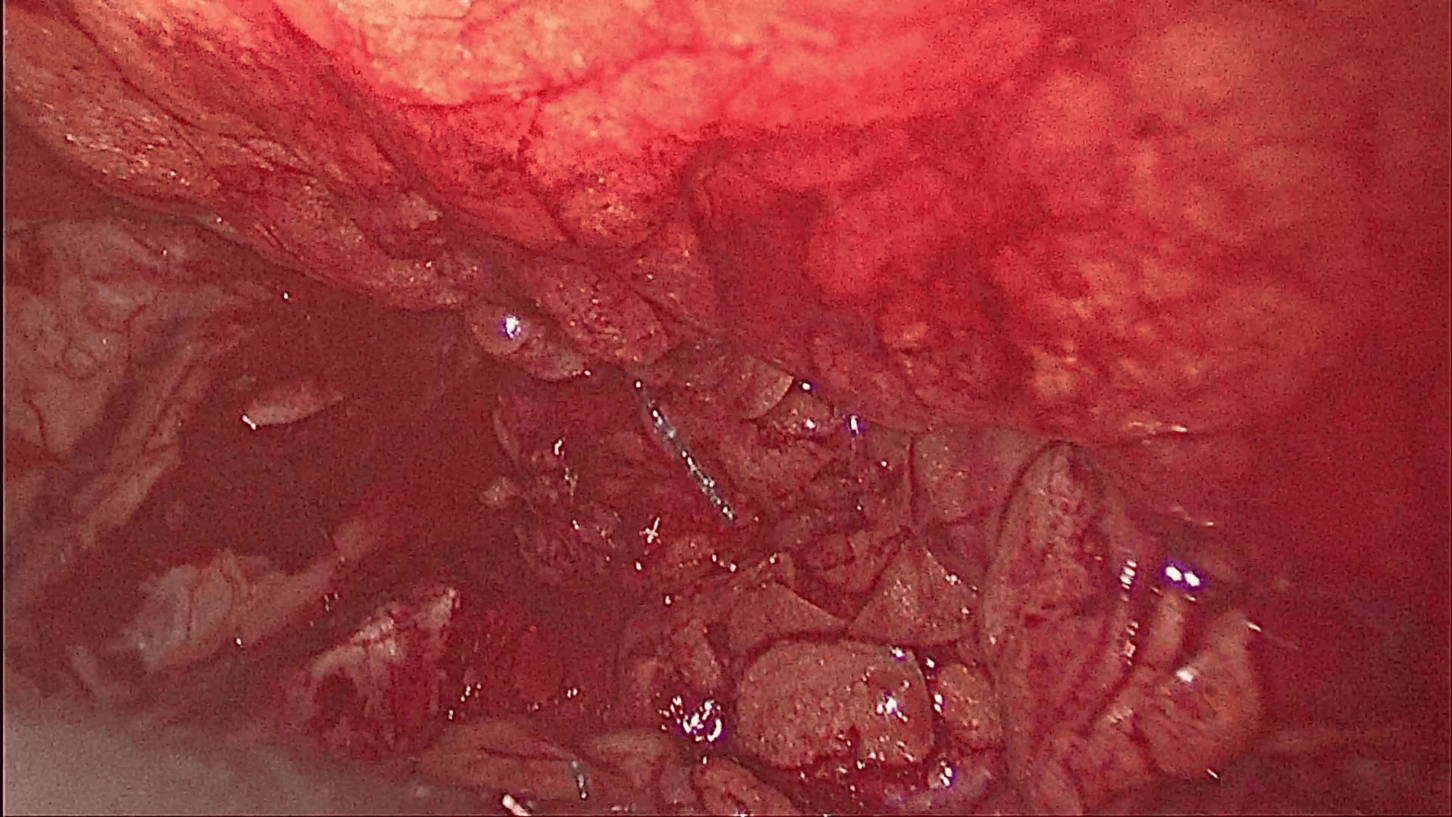
Closed defect.

**Figure 10:**
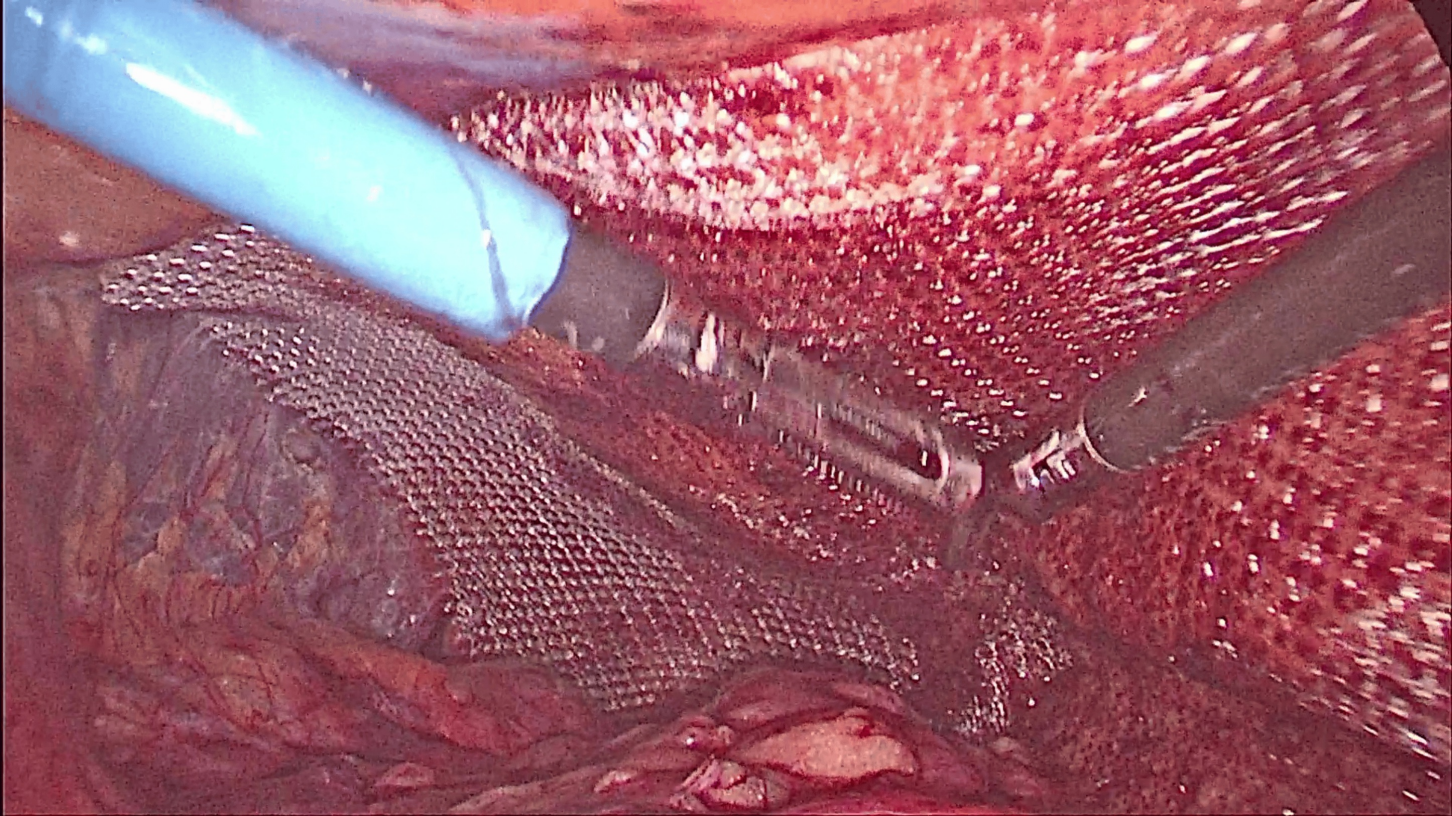
Placing the mesh.

**Figure 11:**
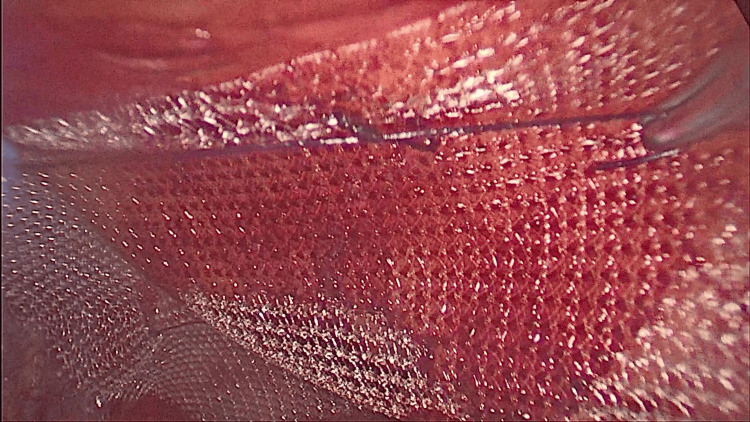
Fixing the mesh.

The defect was closed with absorbable braided sutures to prevent chronic nerve entrapment (Figure 9). A 10 x 15 cm polypropylene mesh trimmed on the sides was introduced and fixed with 3-0 absorbable sutures (Figures 10, 11). The wound was closed after releasing the carbon dioxide (CO2). A detailed video of the surgical procedure is shown in Video 1.

**Video 1:** This comprehensive educational video demonstrates a laparoscopic lumbar hernia repair surgery. (1) Preoperative CT scan and patient positioning. (2) Port placement and dissection techniques. (3) Hernia sac identification and reduction. (4) Mesh placement and fixation. (5) Closure and postoperative picture.

The patient was then changed to a supine position and bilateral TEP inguinal hernia repair was done with new draping and a new set of instruments as a different surgery. The postoperative period was uneventful. He was given three doses of paracetamol infusion postoperatively for analgesia for 36 hours. Since the patient’s bladder was herniating to the base of the scrotum, he was kept on Foley’s catheter for two weeks to avoid bladder atony and possible urinary retention due to subclinical prostatic hypertrophy.

The patient was discharged on postoperative day two and the urinary catheter was removed after two weeks. No evidence of recurrence was found on follow-up for the next 14 months.

## Discussion

Lumbar hernia was first described by Barrett in 1672. Only around 300 cases have been reported till now. Anatomically, three types of lumbar hernia are identified: the superior lumbar hernia, the inferior lumbar hernia, and the diffuse lumbar hernia. Grynfeltt hernia occurs in the superior lumbar triangle, also known as the Grynfeltt-Lesshaft triangle, bordered by the 12th rib and posterior inferior serratus muscle superiorly, laterally by the posterior border of the internal oblique muscle, and medially by the anterior border of the erector spinae muscle [3]. Lumbar hernias can be congenital (18%) or acquired (82%). It can arise spontaneously (54%) or secondarily due to trauma or surgery [4]. This is a rare case report of laparoscopic total extraperitoneal repair of Grynfeltt-Lesshaft hernia simultaneously with TEP repair of bilateral inguinal hernia. The common presenting complaint for lumbar hernia is abnormal swelling. Incarceration and strangulation are seen in 25% and 8%, respectively [5]. Our patient also presented with left loin swelling. Being very rare, lumbar hernia is often misdiagnosed as lipoma and gluteal abscess [6,7]. CT scan is the gold standard for diagnosis [8]. CECT was done in the present case to confirm the diagnosis.

Surgical correction is the treatment of choice, but due to less number of cases and the nonavailability of systematic reviews, the surgical technique is not standardized. Different techniques have been described. Most of the case reports show open hernia repair. Laparoscopic management of these hernias is very challenging. In a retrospective single-center cohort study of 12 patients over seven years, Shadhu et al. described open mesh hernioplasty of upper lumbar hernia [9]. Laparoscopic repair was done only in a few cases compared to open repair. Among these, TAPP repair is more commonly reported. So far, only one case of TEP repair for Grynfeltt-Lesshaft hernia and one case of TEP repair for Petit’s hernia were found in the literature [10,11]. Postema et al. created extraperitoneal space using an inflatable balloon but we have used dissection by zero-degree telescope for the same [10]. Meinke did TEP repair of Petit’s hernia using polytetrafluoroethylene (PTFE) mesh instead of prolene mesh used in the present case. Though being technically simple for a surgeon who does TEP inguinal hernia regularly, it has not been attempted for decades since one case was reported in 2003 [10]. The present case did not have any adverse perioperative outcome and was on regular follow-up for more than one year.

## Conclusions

Laparoscopic TEP repair is a safe and simple minimally invasive procedure for lumbar hernias. It can be attempted by surgeons who are proficient in laparoscopic TEP repair for inguinal hernias and with a sound knowledge of lumbar hernia anatomy. More case reports may be required to develop a standardized surgical technique.
